# Medical News and Miscellany

**Published:** 1890-01

**Authors:** 


### Medical News and Miscellany.

DR. J. H. HARDEE, of Pleasanton, a subscriber of the JOURNAL, is attending a special course at the Cincinnati Polyclinic.

DR. B. E. HADRA, of Galveston, was elected 1st Vice-President of the Southern Surgical and Gynecological Association at a recent meeting of that body.

DRS. R. M. SWEARINGEN, J. M. Litten and W. J. Matthews, of Austin, were elected members of the Board of School Trustees for the city, Dr. Swearingen being chosen President.

MARRIED.—At Hempstead, Texas, January 7, inst., Dr. W. T. Harris to Miss Mae, daughter of Dr. Van B. Thorton of that city. The JOURNAL acknowledges the receipt of an invitation.

Dr. J. A. Denson, of Granger, an old friend of the JOURNAL, met with a serious accident recently; he slipped and fell several feet, fracturing the leg and fearfully lacerating his foot. The JOURNAL extends to him its sincerest sympathy.

THE JOURNAL is pained to chronicle the death of Dr. J. B. Robertson, of Goliad. Dr. Robertson was widely known as Gen. Robertson. He commanded a brigade in the late war. He was an old and honored member of the Texas State Medical Association.

---

DR. B. F. CHURCH has been appointed visiting physician to the Confederate Home at Austin,—a good appointment. By the bye, the Doctor was presented with a valuable horse as a Christmas-gift by an appreciative patient whom the Doctor had "pulled through" by an all night visit, and careful watching. Good!

---

DIED, in Oakland, Texas, December 12, ult., Dr. Thomas Mortimer Laidley, an old and much respected resident of Colorado county, and for thirty-three years a practitioner of that section. The Doctor was born in Barbourville, Va., April 1826, and was a graduate of Jefferson Medical College of the class of 1849.

---

FOR SALE AT A BARGAIN.—A good home and practice; a good five-room dwelling on a four acre lot, well improved, a good kitchen and dining room, a servant's room and good out-houses; fine young orchard and vineyard; good brick cistern; at flourishing railroad depot. Postoffice and express office, also good school. Address, DR. WM. OSBORNE,
Axtell, McLennan county, Texas.

---

MORE PIRACY.—Speaking of "effrontery," the North-Western Medical Journal incorporates in the December number nearly the whole of the original matter published in DANIEL'S TEXAS MEDICAL JOURNAL for that month, and without credit. Cool! It appropriates the excellent article by Dr. Bibb, of Saltillo, "Compound Presentation;" that interesting paper on "Puerperal Eclampsia," by Dr. Kingsley, of San Antonio, read before the West Texas District Medical Society and voted to this, the official organ of the society; and the valuable paper on "Placenta Prævia," by Dr. Wortham: the latter, however, is credited; in regard to the other two, no reference, whatever, is made to the source whence Dr. Fishblatt, the editor, got them. Is this fair? is it honorable journalism?

Dr. R. M. Swearingen delivered the address at the memorial exercises, held in Austin, December 12, on the occasion of the burial of Jefferson Davis. It was a splendid specimen of eloquence and pathos, and did ample justice to the Doctor's reputation as an orator.

Dr. E. P. Becton delivered the address on the same occasion at Sulphur Springs. We acknowledge the receipt of a copy. As usual with all of Dr. Becton's speeches it "rang out like a bell," and was couched in beautiful and touching words.

Dr. A. G. Clopton delivered the memorial address in Jefferson.

These three gentlemen are all ex-Presidents of the Texas State Medical Association, and all well-known and popular orators.

---

An Avalanche of Journals.—Dr. A. L. Hummel, of Philadelphia, is a most indefatigable publisher. We see by the Philadelphia Enquirer, that he has organized a stock company under the name of "The University Press," which company, with Dr. Hummel as general manager, will shortly issue six journals! They are to cover and embrace the entire range of literature. The literary journal is to have an editor-in-chief, not yet chosen, who is to be assisted by Dr. J. S. Billings, Rev. George David Boardman and a host of other geniuses. These several journals will be issued under the auspices of the several departments of the University of Pennsylvania in addition to the two already published by Dr. Hummel in the interest of that Institution.

---

Removals.—Dr. Will B. Davis, whom the members of the Texas State Medical Association will remember as an erstwhile active member, and whose remarkable success with carbolic acid injections into hemorrhoidal tumors created a sensation at the Tyler meeting in 1883, has removed to Colorado for his health and is located at Pueblo, to which point he directs us to send him the Journal, that he may keep pace with his old friends in the Lone Star State. Two years ago Dr. Davis gave up practice and went to St. Louis, where he took charge of Prof. Jno. P.

Bryson's large genito-urinary clinic and assisted the Professor in his surgery, thus fitting himself for this kind of practice, to which the Doctor intends confining himself in future. His old friends will be pleased to hear that Dr. Davis' health is improving, and that he has resumed practice.

Dr. J. O. Lowrie has removed from Timpson to Coleman City —the "Star City of the West."

Dr. J. G. Daniels has removed from Gilmer to Tyler; Dr. J. W. Hamilton from Webberville to Lampasas; Dr. R. A. Taylor from Milwood to Nevada, Collin county.

Dr. W. F. Flewaelen has removed from Ellinger to Belton.

Dr. M. L. Haggard has removed from Midlothian to Haskell City.

Dr. J. A. Gibson has removed from Foster to Richmond, Texas.

In future, Dr. O. M. Conerly will reside at Bevins, Cass county, to which place he has removed from Wayne, same county.

---

DR. F. E. YOAKUM.—Texas is losing some of her best men. Our much esteemed friend and subscriber, Dr. F. E. Yoakum, has left Greenville, Texas, and located at Shreveport, Louisiana. The good people of Bossier Parish are to be heartily congratulated on the accession to the medical ranks of a man of such ability as a practitioner, and sterling worth as a citizen. Moreover, the Doctor is the embodiment of courtesy and geniality. He will be much missed at our annual conventions of the State Medical Association, of which body he is an active and much honored member. He is also a member of the local societies of Hunt county, and was President of the East Line (District) Society, and a member also of the National Medical Association. The Doctor limits his practice to diseases of the eye, ear, nose and throat, and for this specialty he is well fitted. In addition to his regular thorough medical education, he has had the benefit of special courses at Louisville, St. Louis and Chicago. Dr. Yoakum writes us that DANIEL'S TEXAS MEDICAL JOURNAL is very popular in North Louisiana, and as he has become a member of the Shreveport Medical Society, he hopes to send the JOURNAL some good papers. One, by himself, on the use of the microscope in diagnosis, which he read before that Society, he has promised us for an early issue. We take pleasure in commending the Doctor to the brethren of Louisiana as a "first-class" man, in every respect—a physician well worthy of their fullest confidence and esteem.

Dr. F. M. Hicks, of Tyler, a genial gentleman and a staunch friend of the JOURNAL, has also left Texas, but temporarily, we hope. He has gone to San Diego, Cal., for his health. The JOURNAL'S best wishes go with him, and we hope soon to chronicle his recovery and return.

---

THE following letter exhibits such amusing effrontery that we publish it for the information of our exchanges:

J. H. CHAMBERS & Co., Publishers, 914 Locust street,
ST. LOUIS, December 3, 1889.

*Buffalo Medical and Surgical Journal, Buffalo, N. Y.:*

GENTLEMEN—To answer the many requests which have been made by general medical journals to exchange with the Annals of Surgery, and whereas our editor has no use for such journals, we have concluded to mail the Annals of Surgery to such journals, with the understanding that they give editorial notices at least three times a year of the Annals.

Should you desire the Annals of Surgery during next year, kindly inform us, accepting of those conditions, and oblige,

Yours truly, J. H. CHAMBERS & Co."

—Buffalo Medical and Surgical Journal.

We were similarly complimented. It is unnecessary to say we dropped the thing into the waste basket, without reply, and erased the Annals from our "ex"-list. Last year something similar occurred. Mr. Chambers informed all or most of the American journals that the Annals was so "valuable" that he would have to have "boot" in exchanging. There seems to be a prevalent opinion with publishers like Mr. Chambers that no good can come out of the American Nazareth; but anything surgical from "furrin parts" is worth noticing. No matter, if

DANIEL'S TEXAS MEDICAL JOURNAL reports a successful removal of a suppurating kidney by a Texas surgeon, or the successful removal of an immense uterine fibroid by hypodermic injection of ergotine, or the enucleation of a uterine fibroid complicating pregnancy, and at the time of delivery, or any other skillful operation never before or rarely done, the case is not worth incorporating in, or referring to, in Mr. Chambers' Annals. Well, so goes the world; the way Americans toady to the English, French and Germans is disgusting. As we have said before, we are a set of flunkies. In dismissing this subject, we call attention to the *rhethoric* of the above letter and suggest that it would be difficult to *parse* it by any known rules.

Without having, or desiring the benefit of the Annals, we give the above "editorial notice." We cannot promise to give "editorial notices at least three times a year of the Annals," however.

---

THE MEDICAL MIRROR.—We welcome to our exchanges and to the guild, the Medical Mirror, a handsome new journal issued at St. Louis, by Dr. I. N. Love, as per announcement made some months ago. The first number is fully up to our high expectation, and reflects credit alike on the editor and the publishers. It is a royal octavo, double column with fifty-two pages of text and twenty-eight of advertisements. The table of contents embraces a pleasing variety of good reading, while the large and very respectable advertising patronage with which Brother Love begins fairly makes one's eyes dilate, and one's mouth water. The Doctor is the embodiment of enterprise as well as of good taste and good sense. Success to the Mirror.

